# NORRISK 2 score is associated with dementia and MCI—the HUNT study

**DOI:** 10.3389/fnagi.2026.1750636

**Published:** 2026-02-26

**Authors:** Silje Kleven, Linda Ernstsen, Marte Kvello-Alme, Stian Lydersen, Geir Selbæk, Rannveig Sakshaug Eldholm

**Affiliations:** 1 Department of Neuromedicine and Movement Science, Norwegian University of Science and Technology, Trondheim, Norway; 2 Department of Geriatric Medicine, Clinic of Medicine, St. Olavs Hospital, Trondheim, Norway; 3 Department of Public Health and Nursing, Norwegian University of Science and Technology, Trondheim, Norway; 4 Clinic of Medicine, St. Olavs Hospital, Trondheim, Norway; 5 Department of Psychiatry, Levanger Hospital, Nord-Trøndelag Hospital Trust, Levanger, Norway; 6 Department of Neurology, Levanger Hospital, Nord-Trøndelag Hospital Trust, Levanger, Norway; 7 Department of Mental Health, Regional Centre for Child and Youth Mental Health and Child Welfare, Norwegian University of Science and Technology, Trondheim, Norway; 8 Norwegian National Centre for Aging and Health, Vestfold Hospital Trust, Tønsberg, Norway; 9 Institute of Clinical Medicine, University of Oslo, Oslo, Norway; 10 Department of Geriatric Medicine, Oslo University Hospital, Oslo, Norway

**Keywords:** cardiovascular risk score, dementia, HUNT, mild cognitive impairment, NORRISK 2, modifiable risk factors

## Abstract

**Background:**

Cardiovascular disease (CVD) risk factors are associated with the risk of cognitive decline and dementia. Composite CVD risk scores integrate multiple risk factors and may capture the cumulative burden of CVD risk relevant to cognitive outcomes. However, the long-term association between established CVD risk scores and subsequent dementia and mild cognitive impairment (MCI), and potential differences in these associations between males and females, remains insufficiently studied. This study examined the association between NORRISK 2, a CVD risk model estimating 10-year risk of fatal- and non-fatal CVD, and the presence of dementia and mild cognitive impairment (MCI) in males and females, after 22 years of follow-up.

**Methods:**

Participants from The Trøndelag Health Study (HUNT), a longitudinal, population-based health study, were included. NORRISK 2 scores were based on data from HUNT2 (1995-1997). Cognitive status was assessed in the sub-study HUNT4 70+ (2017–2019) and categorized as cognitively unimpaired (CU), MCI, or dementia. We used multinomial logistic regression with NORRISK 2 as the predictor and cognitive status 22 years later as the main covariate.

**Results:**

The study sample consisted of 6,971 participants (57.6% females, mean age at HUNT2 56.1 years). At HUNT4 70+, 14.0% of the participants had developed dementia, and 34.6% had developed MCI. Per one percent increase in NORRISK 2 score, the relative risk of developing dementia increased by 14% for males (relative risk ratio (RRR) = 1.14; 95% CI 1.12–1.17) and 28% for females (RRR = 1.28; 95% CI 1.25–1.31). The relative risk of developing MCI increased by 4% for men (RRR = 1.04; 95% CI 1.02–1.05) and 10% for women (RRR = 1.10; 95% CI 1.08–1.12).

**Conclusion:**

A higher NORRISK 2 score was associated with an increased risk of dementia and MCI in both males and females, with the strongest associations observed in females.

## Introduction

Dementia is a growing global health challenge. It is currently the sixth leading cause of death, and a major contributor to disability and dependency among older people (World Health Organization [WHO], 2021; GBD 2021 Causes of Death Collaborators, 2024). In 2019, an estimated 55 million people were living with dementia worldwide, a number projected to reach 139 million by 2050 (World Health Organization [WHO], 2021). In Norway, prevalence among those 70 years and older is estimated at 14.6%, with projections suggesting a rise from 101,000 cases in 2020 to nearly 237,000 by 2050 (Gjøra et al., 2021).

Mild cognitive impairment (MCI), affecting around 24% of the global population over 60 years (Salari et al., 2025) and 35.3% of Norwegians 70 years and older (Gjøra et al., 2021), is often considered a prodromal stage of dementia. However, MCI is a heterogeneous condition with multiple potential etiologies, including reversible factors such as depression, excessive alcohol intake, or other medical and psychosocial conditions (Petersen et al., 2018). Despite this variability, MCI remains a valuable early indicator of underlying neurodegenerative disease.

Currently there is no effective treatment for dementia, although research in the field is advancing rapidly. Consequently, early identification of individuals at risk and prevention through modifiable risk factors have become a public health priority (Livingston et al., 2024). The Lancet Commission have identified 14 modifiable risk factors that account for approximately 45% of all cases of dementia worldwide (Livingston et al., 2024). Even small delays in disease onset could substantially reduce the burden of dementia at both individual and societal levels (Lewis et al., 2014).

A growing body of evidence shows that cardiovascular (CVD) risk factors play a central role in the development of cognitive decline and dementia. Conditions such as hypertension, hyperlipidemia, diabetes, obesity, smoking and physical inactivity are among risk factors identified by the Lancet Commission. Importantly, many of these factors exert their greatest impact in midlife, long before clinical symptoms occur (Livingston et al., 2024). Moreover, these risk factors often co-occur and interact with each other, making it difficult to isolate individual effects.

Consequently, composite measures of cardiovascular health have been developed to capture the combined impact of these factors. And several of these have also been associated with cognitive outcomes, such as the Cardiovascular Risk Factors, Aging and Dementia (CAIDE) dementia risk score (Kivipelto et al., 2006; Fayosse et al., 2020), the Lifestyle for Brain Health (LIBRA) index (Schiepers et al., 2018; Sewell et al., 2026) and the Updated Lifestyle for Brain Health (LIBRA2) score (Rosenau et al., 2025), the Systematic Coronary Risk Evaluation 2 (SCORE2) (Conroy et al., 2003; SCORE2 working group and ESC Cardiovascular risk collaboration, 2021; Zheng et al., 2023), the Framingham Risk Score (D’Agostino et al., 2008; Fayosse et al., 2020) and the Framingham Stroke Risk Profile (D’Agostino et al., 1994; Yuan et al., 2024), and composite indices such as Life’s Simple 7 (LS7) (Lloyd-Jones et al., 2010; Huang et al., 2026) and the updated Life’s Essential 8 (LE8) (Lloyd-Jones et al., 2022; Wang et al., 2024). A recent Norwegian study comparing multiple cardiovascular and lifestyle-based risk models (including CAIDE, ANU-ADRI, CogDrisk, LIBRA and LIBRA2) reported that all models were associated with dementia (Stubs et al., 2025). However, the same study concluded that none of the models performed better than a simple model including age and education alone (Stubs et al., 2025). And although many of these tools show statistical associations with cognitive decline or dementia across different populations, no single model has emerged as a gold standard for dementia risk assessment (Mohanannair Geethadevi et al., 2023). Taken together, these findings support the overarching concept that vascular and cardiometabolic risk factors contribute to cognitive decline and dementia, even though the optimal way to operationalize the risk remains unclear.

Building on this evidence, the present study uses a cardiovascular risk score developed and validated for use in the Norwegian population as a proxy for overall cardiovascular health. The NORRISK 2 score is a risk model developed and validated to predict 10-year risk of fatal- and non-fatal incident acute myocardial infarction or cerebral stroke in individuals without known cardiovascular disease (Selmer et al., 2020). The score comprises seven metrics (age, systolic blood pressure, use of antihypertensive medication, total cholesterol, HDL-cholesterol, current smoking status, and family history of early coronary heart disease), and the result is expressed as a percentage representing the estimated risk of experiencing the outcome.

Current prevention guidelines emphasize targeting risk factors during midlife (Livingston et al., 2024). Studies aiming to identify early predictors of cognitive decline must therefore have sufficiently long follow-up periods to include the onset of dementia or MCI, which can occur decades after initial risk exposure (Jia et al., 2024). The HUNT Study is a large population-based cohort with up to 37 years of follow-up, and offers a unique opportunity to explore these associations (Holmen et al., 1990; Holmen et al., 2003; Krokstad et al., 2013; Krokstad et al., 2022; Åsvold et al., 2023; Næss et al., 2024).

In recent years, sex differences in cognitive aging have gained increasing attention, with evidence pointing to distinct patterns in the development and progression of dementia and MCI between men and women. Although females are more likely to develop Alzheimer’s disease (Alzheimer’s Association, 2023), they often perform better on episodic memory tasks and show slower age-related memory decline, particularly in earlier stages such as MCI (Anstey et al., 2021; Bloomberg et al., 2021). This paradox has led researchers to investigate whether modifiable risk factors may affect males and females differently. One such study found that the number of risk factors present moderates sex differences in memory performance (LaPlume et al., 2022). Among individuals with few risk factors, men showed greater age-related memory decline than women. But among those with multiple risk factors, both sexes showed similar decline, and the female memory advantage disappeared (LaPlume et al., 2022). These findings underscore the importance of considering sex-specific pathways in dementia risk, particularly in relation to CVD health.

In this study, we used data from the HUNT Study to examine whether the NORRISK 2 score is associated with dementia and MCI in males and females after a 22-year follow-up period. Using a risk score developed and validated for the Norwegian population within a large Norwegian cohort provides a contextually relevant and population-appropriate measure for examining the longitudinal associations between cardiovascular burden and both dementia and mild cognitive impairment.

## Materials and methods

### Study design and participants

Data have been collected as part of The HUNT Study. Details on The HUNT Study have been provided previously (Holmen et al., 1990; Holmen et al., 2003; Krokstad et al., 2013; Krokstad et al., 2022; Brumpton et al., 2022; Åsvold et al., 2023). In brief, the entire adult population (20 years and older) living in the former county of Nord-Trøndelag, Norway, has been invited to participate in four consecutive surveys: HUNT1 (1984–1986), HUNT2 (1995–1997), HUNT3 (2006–2008), and HUNT4 (2017–2019). The HUNT surveys include questionnaires, interviews, clinical examinations, and collection of biological samples, and have a high participation rate ranging from 54 to 89% (Brumpton et al., 2022). Nord-Trøndelag consists mainly of rural areas and small towns, and is quite representative of the overall Norwegian population, except for the lack of large cities and a lower number of immigrants and people with higher education (Åsvold et al., 2023). In the HUNT4 Survey, all participants 70 years and older were further invited to participate in the sub-study HUNT4 70+, where they underwent a cognitive assessment (Skjellegrind et al., 2025). Out of 19,403 eligible individuals, 9,956 (51.3%) consented to participation and were included in HUNT4 70+ (Skjellegrind et al., 2025).

**FIGURE 1:**
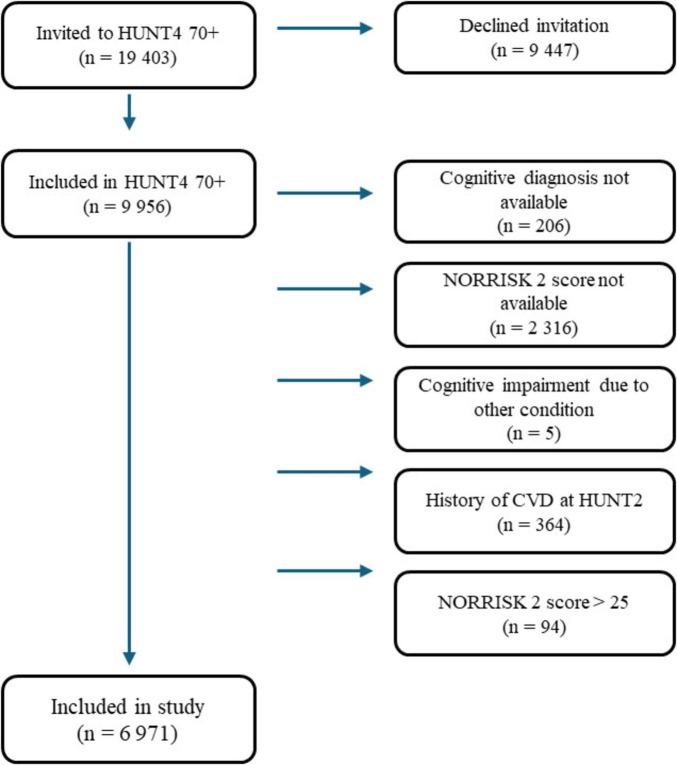
Flow chart of included study population. CVD, cardiovascular disease.

In this study, we included participants with available data for calculating a NORRISK 2 score at HUNT2 and with a cognitive diagnosis (cognitively unimpaired (CU), MCI, or dementia) from HUNT4 70+. HUNT2 was selected as the baseline since it was the earliest HUNT survey to capture all risk factors required for the NORRISK 2 score. Five participants had cognitive impairment caused by depression and were excluded. The NORRISK 2 score is intended for individuals without prior CVD, and according to the NORRISK 2 protocol, participants with previous myocardial infarction, angina pectoris, or stroke (*n* = 364) were excluded. There were very few participants with a NORRISK 2 score above 25% (*n* = 94); these were therefore considered as outliers and excluded. In total, 6,971 individuals were included in the final study (Figure 1).

### Assessment of NORRISK 2

Participants’ NORRISK 2 score at the time of participation in HUNT2 was estimated based on age, systolic blood pressure, use of antihypertensive medication, total cholesterol, HDL-cholesterol, current smoking status, and family history of early coronary heart disease (Selmer et al., 2020). Information on smoking, family history, and the use of antihypertensives was self-reported through questionnaires. Systolic blood pressure was measured in the sitting position according to standardized methods (Holmen et al., 2003). Three consecutive automatic oscillometric blood pressure measurements (Dinamap Critikon model 845XT or Dinamap XL model 9301) were recorded at a 1-min interval, and the mean of the second and the third readings was calculated and reported (Gabin et al., 2017). Total cholesterol and High-density lipoprotein (HDL) cholesterol were measured in non-fasting serum. Total cholesterol was defined as a continuous variable while HDL cholesterol was defined as a binary variable (low HDL cholesterol yes/no) with a cutoff of <1.3 mmol/L in women and < 1.0 mmol/L in men. Observational studies have shown that low HDL cholesterol is a risk factor for CVD, but high HDL cholesterol is not necessarily a protective factor (Siddiqi et al., 2015). Therefore, in the NORRISK 2 score, HDL cholesterol was dichotomized into low HDL cholesterol versus high HDL cholesterol, according to the definition of the metabolic syndrome (Alberti et al., 2009). NORRISK 2 score was used as a summary measure of cumulative vascular risk, integrating established CVD risk factors into a standardized, population-validated score.

### Assessment of cognitive status

In HUNT4 70+, participants were interviewed about self-perceived cognitive function and underwent cognitive assessment at a field station (85.6%), in the participant’s home (8.1%), or at the nursing home where the participant was residing (6.3%) (Skjellegrind et al., 2025). All participants interviewed at the field station or at home were assessed using the same protocol, which covered cognition, daily life function, neuropsychiatric symptoms, subjective cognitive decline, symptom onset, and symptom course (Skjellegrind et al., 2025).

The cognitive assessment protocol included the Montreal Cognitive Assessment (MoCA) scale, a global cognitive test with scores 0–30, higher scores indicating better cognition (Nasreddine et al., 2005). In cases with a MoCA score of 22 or higher, further testing with the Word List Memory Task (WLMT), immediate and delayed recall, from the Consortium to Establish a Registry of Alzheimer’s disease (Morris et al., 1989) was conducted. This was done to increase sensitivity for detecting subtle memory impairments among participants with relatively preserved MoCA performance. If a participant screened positive for possible cognitive impairment, either based on subjective cognitive decline, poor results on cognitive tests, or being a nursing home resident, a close relative was contacted for additional information on cognitive function, neuropsychiatric symptoms, function in daily life, and symptom onset and course (Gjøra et al., 2021).

For nursing home residents, a protocol adapted to the participant’s functional level was applied (Skjellegrind et al., 2025). Information on the participants’ cognition and dementia symptoms was provided by health personnel who knew the participant well. In cases where the participant was considered to have moderate or severe dementia, the Severe Impairment Battery-8 (Schmitt et al., 2013) was used instead of MoCA.

Dementia diagnoses were based on DSM-5 diagnostic criteria (American Psychiatric Association, and Dsm-5 Task Force, 2013). For each participant, two research and clinical experts, from a pool of nine, reviewed all available information and independently made a cognitive diagnosis. If no consensus was reached, a third expert was consulted. Participants were classified into one of the following categories: no cognitive impairment, amnestic MCI, non-amnestic MCI and dementia (Skjellegrind et al., 2025). In this study, participants were classified into three groups: cognitively unimpaired, MCI, or dementia.

### Assessment of covariates

Covariates were included to test the robustness of the association, based on prior literature and clinical relevance (Livingston et al., 2024; Global Burden of Cardiovascular Diseases and Risks 2023 Collaborators, 2025). Included covariates were age, education, marital status, body mass index (BMI), diabetes, physical inactivity, alcohol use, depressive symptoms, and Apolipoprotein E (ApoE) ε4-status. Age was computed based on birth date and participation date, rounded to one decimal. Marital status (married or not) was obtained from the Norwegian National Population Register, a national register containing information about everyone who resides or has resided in Norway. Data on education (highest level of education achieved: primary, high school, college or university < 4 years, college or university ≥ 4 years), diabetes (yes/no), and alcohol consumption (total units per week) were self-reported through questionnaires. Physical activity (physically active/physically inactive) was categorized based on World Health Organization (WHO) recommendations (World Health Organization [WHO], 2020). In The HUNT2 Survey, participants were asked to report average hours of low-intensity physical activity per week and average hours of vigorous-intensity physical activity per week in the last year (Kurtze et al., 2007). Participants were dichotomized into physically active (meeting the recommendations of intensity and duration from WHO and physically inactive (not meeting the recommendations of intensity and duration from WHO). Although the categorization in this study is based on the 2020 WHO guidelines, which recommend at least 150–300 min of moderate-intensity, or 75–150 min of vigorous-intensity physical activity per week for optimal health (World Health Organization [WHO], 2020), these thresholds are also broadly consistent with earlier recommendations from the 1990s, during which adults were advised to engage in at least 30 min of moderate-intensity physical activity on most days of the week, corresponding to approximately 210 min per week (Pate et al., 1995). Thus, the physical activity variable used in this study also reflects recommendations for physical activity from the time of data collection. Depressive symptoms were measured using the depression subscale from the Norwegian version of the Hospital Anxiety and Depression Scale (HADS), a widely used screening tool for symptoms of anxiety and depression in population-based studies (Mykletun et al., 2001; Bjelland et al., 2002). In this study, only the depression subscale was used. The HADS subscale for depression consists of 7 items, each scored from 0 to 3, resulting in a total score ranging from 0 to 21. The score was treated as a continuous variable, with higher scores indicating more severe depressive symptoms (Mykletun et al., 2001; Bjelland et al., 2002). BMI was calculated based on height and weight measurements. Height and weight were measured with participants wearing light clothes without shoes. Height was given in whole centimeters, and weight was given in kilos, rounded to the nearest half kilo (Holmen et al., 2003). All covariates, except for ApoE ε4 status, were measured in the HUNT2 Survey.

ApoE ε4 status was obtained from genome-wide genetic data generated within the HUNT Study (Brumpton et al., 2022). In total, DNA from 71,860 participants has been genotyped in the HUNT Study, and ApoE ε4 allele status is determined by two single-nucleotide polymorphisms (SNPs): rs7412 and rs429358 (Brumpton et al., 2022). In this study, participants were classified as non-carriers, heterozygote carriers, or homozygote carriers of the ApoE ε4-allele.

An additional variable, ethnicity, was included descriptively in the baseline table. Country of residence at age one was used as a proxy for ethnicity, as no direct measure was available in HUNT.

### Statistical analysis

Descriptive statistics are reported as mean and standard deviation (SD) for continuous variables and counts and percentages for categorical variables (Table 1).

**TABLE 1:** Descriptive statistics of study population (*n* = 6,971).

Characteristics	Total sample	Males	Females	Missing
Participants, n (%)	6,971 (100)	2,958 (42.4)	4,013 (57.6)	0
Age, mean (SD)	56.07 (6.3)	55.4 (5.8)	56.5 (6.6)	0
Norwegian ethnicity, n (%)	6,402 (98.7)	2,659 (99.0)	3,743 (98.5)	484
Education				168
Primary school	2,805 (41.2)	913 (31.4)	1,892 (48.6)	
High school	2,407 (35.4)	1,200 (41.2)	1,207 (31.0)	
College/university < 4 years	849 (12.5)	406 (13.9)	443 (11.4)	
College/university > 4 years	742 (10.9)	393 (13.5)	349 (9.0)	
Married, n (%)	5,734 (82.3)	2,522 (85.3)	3,212 (80.1)	7
NORRISK 2 score, mean (SD)	6.4 (5.0)	8.4 (5.0)	4.9 (4.3)	0
NORRISK 2 score components:				
SysBP (mmHg), mean (SD)	138.1 (18.6)	138.7 (16.6)	137.6 (19.9)	0
Hypertension treatment, n (%)	645 (9.3)	227 (7.7)	418 (10.4)	0
Total cholesterol (mmol/L), mean (SD)	6.3 (1.1)	6.1 (1.0)	6.4 (1.2)	0
HDL cholesterol (mmol/L), mean (SD)	1.5 (0.4)	1.3 (0.4)	1.6 (0.4)	0
Smoking, n (%)	1,375 (19.7)	574 (19.4)	801 (20.0)	0
Heredity to CVD, n (%)	1,261 (18.1)	474 (16.0)	787 (19.6)	0
BMI (kg/m^2^), mean (SD)	26.8 (3.7)	26.7 (3.0)	26.8 (4.1)	5
Diabetes, n (%)	133 (1.9)	67 (2.3)	66 (1.6)	6
Physically inactive, n (%)	2,833 (43.4)	1,064 (37.2)	1,769 (48.3)	448
Alcohol (units/week), mean (SD)	1.7 (2.2)	2.3 (2.6)	1.1 (1.7)	383
HADS-D scale, mean (SD)	3.7 (3.0)	3.9 (2.9)	3.6 (3.0)	418
ApoE ε4-allele, n (%)				41
Non-carrier	4,864 (70.2)	2,061 (70.1)	2,803 (70.3)	
Heterozygote carrier	1,884 (27.2)	806 (27.4)	1,078 (27.0)	
Homozygote carrier	182 (2.6)	73 (2.5)	109 (2.7)	

SysBP, Systolic blood pressure; HDL cholesterol, High-density lipoprotein cholesterol; CVD, Cardiovascular disease; BMI, Body mass index; HADS-D scale, Hospital Anxiety and Depression Scale—Depression subscale; ApoE ε4, Apolipoprotein E ε4; SD, Standard deviation.

Cognitive status was defined as a three-category variable with these categories: cognitively unimpaired (CU), MCI, and dementia (all-cause dementia). NORRISK 2 score was defined as a continuous variable with possible values ranging from 0 to 100%, where higher values reflect the level of predicted CVD risk for which the score was originally developed.

We used multinomial logistic regression with cognitive status at HUNT4 70+ as the dependent variable and NORRISK 2 score as the independent variable. Main analyses were conducted without adjustment for other covariates, as such adjustment would alter the structure and interpretation of the risk model itself. Next, we conducted analyses with the NORRISK 2 score and one of the following covariates at a time: age, education, marital status, BMI, diabetes, physical inactivity, alcohol use, depressive symptoms, and ApoE ε4-status. A fully adjusted multinomial model could not be estimated because substantial covariate missingness and perfect prediction led to sparse-data and non-convergence.

**FIGURE 2:**
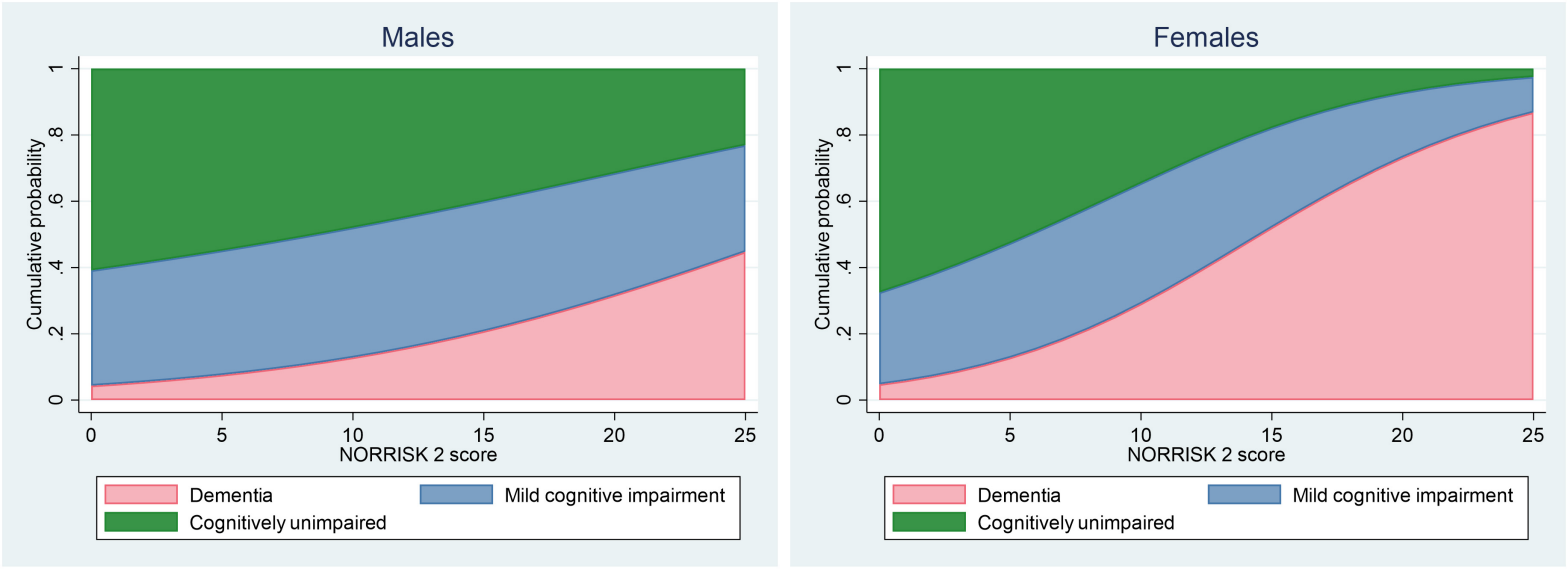
Stacked area plot illustrating cumulative probability of dementia, mild cognitive impairment, and cognitively unimpaired by NORRISK 2 score for males and females. For any given NORRISK 2 score, the height of the pink, blue and green area represents the estimated probability of dementia, mild cognitive impairment and cognitively unimpaired.

To illustrate how the estimated probability of each outcome varies across the range of NORRISK 2 score, we created stacked area plots based on model-estimated probabilities from the unadjusted models (Figure 2). Potential non-linear effects of the NORRISK 2 score were examined using fractional polynomials.

Missing values were handled using available case analysis, that is, in each analysis, cases with data on the relevant variable were included. This was considered adequate, since age was the only covariate that changed the results notably, and age had no missing values. Relative risk ratios (RRR) were reported per 1% change on the NORRISK 2 score.

Analyses were conducted separately for males and females.

Ninety-five percent confidence intervals (95% CI) were reported where relevant, and a two-sided *p* < 0.05 was considered to represent statistical significance.

All statistical analyses were conducted using STATA version 19.5 (StataCorp, 2025).

### Sensitivity analyses

A sensitivity analysis with the inclusion of all participants, including those with a NORRISK 2 score above 25, was conducted. Given the potential for age to influence both NORRISK 2 scores and risk of dementia and MCI, a second sensitivity analysis with four predefined age groups was conducted (45–54, 55–64, 65–74, and 75–84). In addition, since the score is intended for prevention in midlife, we conducted a third sensitivity analysis restricting the study population to participants who were 60 years or younger at the time of NORRISK 2 calculation at HUNT2. This age threshold was chosen to reduce the potential for reverse causation, as individuals closer to the typical onset age for dementia may already exhibit early cognitive changes that could influence risk score components.

### Ethics

This study was approved by the Regional Committee for Medical and Health Research Ethics in Norway (REK Midt 405120). Storage and use of data follow the General Data Protection Regulation (GDPR). Participation in the HUNT Studies is based on written informed consent.

## Results

In total, 6,971 participants (57.6% females) were included in the study. The mean age at HUNT2 was 55.4 years for males (range 47–78, SD 5.8 years) and 56.5 years for females (range 47–82, SD 6.6 years). The study population was ethnically homogenous; among participants with available data, 98.7% reported Norwegian ethnicity. The males in the study had a mean NORRISK 2 score of 8.4% (range 0.9–25.0, SD 5.0) at HUNT2, while the females had a mean NORRISK 2 score of 4.9% (range 0.5–24.8, SD 4.3). Descriptive statistics of the study population at baseline (HUNT2) are presented in Table 1.

At HUNT4 70+, 12.2% of all males and 15.4% of all females had dementia, and 37.9% of males and 32.2% of females had MCI. Table 2 shows absolute numbers and RRR with 95% CI for MCI and dementia for males and females. Results are presented unadjusted and adjusted for covariates separately.

**TABLE 2:** Multinomial logistic regression with three-category cognitive status as the dependent variable and NORRISK 2 score as the main covariate. Relative risk ratios with 95% confidence intervals are presented unadjusted and adjusted for covariates separately.

Model		Males							Females							
	No.		MCI			Dementia			No.		MCI			Dementia		
	Total	Cases MCI/ dementia	RRR	95% CI	*p*-value	RRR	95% CI	*p*-value	Total	Cases MCI/ dementia	RRR	95% CI	*p*-value	RRR	95% CI	*p*-value
**Unadjusted:**	2,958	1,122/361	1.036	1.019–1.054	< 0.001	1.141	1.117–1.166	< 0.001	4,013	1,291/617	1.100	1.077–1.121	< 0.001	1.282	1.254–1.311	< 0.001
**Adjusted for covariates:**																
Age	2,958	1,122/361	1.027	1.006–1.048	0.012	1.049	1.018–1.080	0.002	4,013	1,291/617	1.095	1.065–1.125	< 0.001	1.119	1.083–1.157	< 0.001
Education	2,912	1,103/350	1.026	1.009–1.043	0.003	1.124	1.099–1.149	< 0.001	3,891	1,242/562	1.074	1.052–1.097	< 0.001	1.246	1.217–1.275	< 0.001
Marital status	2,956	1,122/359	1.036	1.019–1.053	< 0.001	1.143	1.118–1.167	< 0.001	4,008	1,287/616	1.098	1.076–1.120	< 0.001	1.279	1.251–1.308	< 0.001
BMI	2,956	1,121/360	1.036	1.019–1.053	< 0.001	1.140	1.116–1.165	< 0.001	4,010	1,290/616	1.097	1.075–1.119	< 0.001	1.278	1.250–1.307	< 0.001
Diabetes	2,956	1,122/361	1.036	1.019–1.053	< 0.001	1.141	1.117–1.165	< 0.001	4,009	1,290/616	1.098	1.077–1.120	< 0.001	1.281	1.253–1.310	< 0.001
Physically inactive	2,857	1,067/337	1.034	1.017–1.052	< 0.001	1.141	1.116–1.166	< 0.001	3,666	1,169/495	1.082	1.059–1.106	< 0.001	1.276	1.245–1.307	< 0.001
Alcohol	2,819	1,067/334	1.035	1.018–1.053	< 0.001	1.142	1.117–1.167	< 0.001	3,765	1,194/570	1.097	1.075–1.120	< 0.001	1.270	1.241–1.299	< 0.001
HADS-D scale	2,817	1,066/338	1.035	1.017–1.053	< 0.001	1.137	1.112–1.162	< 0.001	3,736	1,194/545	1.091	1.068–1.114	< 0.001	1.281	1.251–1.312	< 0.001
ApoE ε4 status*	2,940	1,113/360	1.036	1.019–1.053	< 0.001	1.142	1.118–1.167	< 0.001	3,990	1,283/609	1.099	1.077–1.121	< 0.001	1.291	1.262–1.321	< 0.001

MCI, Mild cognitive impairment; BMI, Body mass index; HADS-D scale, Hospital Anxiety and Depression Scale—Depression Subscale; ApoE ε 4, Apolipoprotein E ε 4; RRR, Relative risk ratios; 95% CI = 95% confidence intervals.

*Adjusted for ApoE ε4 status using three categories: non-carriers, heterozygote carriers, and homozygote carriers.

In unadjusted analyses, there was a clear association between NORRISK 2 score and dementia in both sexes. Although mean NORRISK 2 scores were higher in men, the associations between NORRISK 2 and dementia and MCI were steeper in women, indicating a stronger relative risk per unit increase in the score. For a one-percent increase in NORRISK 2, the relative risk of developing dementia (vs. cognitively unimpaired) increased by 14% for males (RRR = 1.14; 95% CI 1.12–1.17) and 28% for females (RRR = 1.28; 95% CI 1.25–1.31). Adjustments for other covariates showed that age was the only covariate that altered the association between NORRISK 2 and dementia notably. When adjusting for age, the strength of the association was weakened from 14% to 5% for males and from 28% to 12% for females, suggesting that a large part of the association is due to the age component in the NORRISK 2 score, or possibly also that age may act as a confounder or effect modifier in this relationship. Adjustment for education did not meaningfully influence the association. Other covariates similarly had little impact on the association.

The NORRISK 2 score was also associated with MCI, although with a more modest effect size compared to dementia. For each one-percent increase in the NORRISK 2 score, the relative risk of having MCI (vs. cognitively unimpaired) increased by 4% for males (RRR = 1.04; 95% CI 1.02–1.05) and 10% for females (RRR = 1.10; 95% CI 1.08–1.12). Adjustment for covariates did not result in a significant change in the effect estimates for any of the covariates.

The stacked area plots demonstrated marked sex differences in the predicted distribution of outcomes across the NORRISK 2 scale. For females, the curve showed a markedly steeper trajectory, particularly between scores of 10% and 20%, compared to males, indicating a sharper rise in dementia probability with higher cardiovascular risk burden (Figure 2). Investigation of any potential non-linear effects using fractional polynomials did not result in notably different results, data not shown.

### Sensitivity analyses

Sensitivity analyses that included participants with NORRISK 2 scores above 25% yielded similar results to the primary analyses; the associations remained statistically significant with only a slight reduction in effect sizes (see Table 3).

**TABLE 3:** Multinomial logistic regression with three categories of cognitive status as dependent variable and NORRISK 2 score as covariate. Participants with NORRISK 2 score > 25 included.

Males								Females							
		MCI			Dementia					MCI			Dementia		
Tot. No.	Cases MCI/ dementia	RRR	95% CI	*p*-value	RRR	95% CI	*p*-value	Tot. No.	Cases MCI/ dementia	RRR	95% CI	*p*-value	RRR	95% CI	*p*-value
3,022	1140/386	1.030	1.015–1.045	< 0.001	1.119	1.099–1.139	< 0.001	4,043	1299/636	1.100	1.079–1.121	< 0.001	1.261	1.235–1.288	< 0.001

MCI, mild cognitive impairment; RRR, relative risk ratio; 95% CI = 95% confidence interval.

To explore potential age-related differences in the association, sensitivity analyses of four predefined age groups (45–54, 55–64, 65–74, and 75–84) were conducted (see Table 4). Statistically significant associations for dementia were observed in the two youngest age groups (45–54 and 55–64) among males, and in the three youngest age groups (45–54, 55–64, and 65–74) among females. The associations were strongest in the youngest age group for males and in the two youngest age groups for females. For MCI, a significant association was found only in the youngest group (45–54) for males, while for females, a significant association was found in the three youngest groups (45–54, 55–64, and 65–74), with the highest effect size in the youngest group. In the oldest group (75–84), sample sizes for both sexes were too small to conduct meaningful analyses.

**TABLE 4:** Multinomial logistic regression with three categories of cognitive status as dependent variable and NORRISK 2 scores as covariate, separately for four predefined age-groups.

Age	Males								Females							
			MCI			Dementia					MCI			Dementia		
	Tot. No.	Cases MCI/ dementia	RRR	95% CI	*p*-value	RRR	95% CI	*p*-value	Tot. No.	Cases MCI/ dementia	RRR	95% CI	*p*-value	RRR	95% CI	*p*-value
45–54	1,653	634/110	1.048	1.020–1.077	0.001	1.084	1.035–1.135	0.001	2,020	684/96	1.091	1.042–1.141	< 0.001	1.180	1.090–1.277	< 0.001
55–64	1,060	402/151	1.009	0.980–1.040	0.546	1.046	1.005–1.087	0.026	1,470	451/262	1.075	1.034–1.116	< 0.001	1.183	1.135–1.232	< 0.001
65–74	237	83/96	0.932	0.858–1.013	0.097	1.038	0.959–1.123	0.352	485	146/231	1.073	1.009–1.141	0.024	1.106	1.045–1.171	0.001
75–84	8	3/4	1.535	0.327–7.213	0.587	1.229	0.277–5.455	0.786	38	10/28	–	–		–	–	–

MCI, mild cognitive impairment; No, number; RRR, relative risk ratio; 95% CI = 95% confidence interval.

In sensitivity analyses where the study population was restricted to participants 60 years or younger at HUNT2, the associations remained statistically significant for both dementia and MCI for both males and females. Also here, the strongest associations were found among females (see Table 5).

**TABLE 5:** Multinomial logistic regression with three categories of cognitive status as dependent variable and NORRISK 2 scores as main covariate, restricted to participants 60 or younger at HUNT2.

	Males							Females							
		MCI			Dementia					MCI			Dementia		
Tot. no.	Cases MCI/ dementia	RRR	95% CI	*p*-value	RRR	95% CI	*p*-value	Tot. No.	Cases MCI/ dementia	RRR	95% CI	*p*-value	RRR	95% CI	*p*-value
2,433	944/202	1.034	1.013–1.056	0.001	1.073	1.039–1.109	< 0.001	3,030	992/234	1.075	1.040–1.110	< 0.001	1.240	1.188–1.294	< 0.001

MCI, mild cognitive impairment; RRR, relative risk ratios; 95% CI, 95% confidence interval.

## Discussion

Using data from The HUNT2 Survey and The HUNT4 70+ sub-study we investigated whether the CVD risk score NORRISK 2 were associated with dementia and MCI after a 22-year follow-up period. Our results showed that a higher NORRISK 2 score was associated with increased risk of developing dementia. A weaker association was observed for MCI. The associations were evident in both men and women, with the strongest associations being found for women.

To the best of our knowledge, no previous studies have investigated the prospective relationship between NORRISK 2 score and risk of dementia and MCI. However, our findings align with a broader body of evidence showing that several other cardiovascular risk scores and health indices are associated with later-life cognitive decline and dementia (Zheng et al., 2023; Vishwanath et al., 2024; Harrison et al., 2025; Yuan et al., 2024; Song et al., 2020). Together, these studies underscore the importance of cardiovascular health across the life course as a determinant of cognitive aging, supporting the plausibility of the association observed in our analyses.

Previous research have shown that the link between cardiovascular health and cognitive outcomes may differ between populations. In a study comparing associations between cardiometabolic risk and cognitive function in the USA and China, researchers found a significant association in the American population, but these findings were not replicated when studying a Chinese population (Wu et al., 2024). These results imply that there may be regional or genetic differences and highlight the importance of conducting research across diverse populations. A recent comparative study of several CVD-related dementia risk models, also using HUNT data, showed that these scores were associated with dementia, but did not outperform a simpler model including only age and education. Our findings complement this work by evaluating the association between the NORRISK 2 score, and dementia and MCI in Norway. In contrast to the previous study, we draw on a longer follow-up period and additionally assess MCI, allowing us to capture both early and later stages of cognitive decline.

Moreover, our study revealed a stronger association between the CVD risk score and both dementia and MCI in females compared to males. Similar sex-specific patterns have also been reported in other studies (Yuan et al., 2024; Vishwanath et al., 2024). This may suggest that females are more vulnerable to the cognitive consequences of CVD risk factors, potentially due to hormonal influences, differences in vascular physiology, or differences in exposure to risk factors. However, given that females have a higher lifetime risk of dementia, it can also be attributed to survival bias. Further research is needed to clarify the underlying mechanisms and to explore whether sex-specific preventive strategies should be implemented.

In the adjusted models, age was the only covariate that altered the association between the NORRISK 2 score and dementia and MCI significantly, highlighting its potential role as a key confounder. This is a plausible finding, as age is a well-established risk factor for both CVD and cognitive impairment, in addition to influencing both the components in the NORRISK 2 score and the NORRISK 2 score itself. These findings were further supported by age-separated sensitivity analyses. Although the overall analyses showed relatively strong associations between the NORRISK 2 score and the outcomes, age-separated analyses showed more modest effect sizes. Performing separate analyses per age group means that we largely adjust for age. Hence, as expected, the RRRs in these separate analyses are lower than the unadjusted RRRs in Table 2 and closer to the RRRs adjusted for age in Table 2. In both males and females, associations were stronger in the younger groups compared to the older age groups, suggesting that death as a competing risk may be affecting the results. These findings further highlight the importance of considering both sex and age when assessing long-term dementia risk. Although Nord-Trøndelag has a lower number of people with higher education than the national average, this difference did not appear to bias our estimates, as adjustment for education did not materially change the association. The overall minimal impact of other covariates suggests that the observed associations are relatively robust across different subgroups, while the effect of age and sex should be carefully considered in future use or modifications of the risk score.

The inclusion of participants with extreme values of the NORRISK 2 (NORRISK 2 > 25) confirmed the robustness of the primary findings, with only a modest reduction in effect size (0.5–2.2%). This indicates that the exclusion of extreme values in the main analyses did not alter the observed results and supports the validity of the risk score across a broader range of exposure levels. However, the small decrease in effect size may result from the presence of outliers or greater variability at the extremes, which should be considered in future use or validations of the score.

This study has several strengths. It has a large sample size and a population-based inclusion of both home-dwelling and institutionalized participants, a combination that is relatively rare in this field. It has a long follow-up period of 22 years, which is especially important given the long preclinical phase of dementia. Cognitive diagnoses are based on standardized criteria, and cognitive assessments were performed consistently throughout the entire sample, including a consensus process by a clinical expert panel. This is a strength compared to studies using less rigorous registry-based diagnoses. In addition, access to most of the known risk factors for dementia in the dataset reduces the likelihood of residual confounding and strengthens the validity of the observed observation.

However, some limitations must be acknowledged. First, the reliance on self-reported data in some of the components of the NORRISK 2 score may have introduced bias. Second, since cognitive status is only measured in HUNT4 70+, we only know the proportion of participants with dementia and cognitive impairment at that specific time point, but not when they developed the condition, nor whether the NORRISK 2 score at HUNT2 could be affected by a potential preclinical stage of dementia. Consequently, we cannot guarantee that our findings are free from reverse causation. Dementia develops over a long prodromal period during which blood pressure and metabolic parameters may begin to decline (Peters et al., 2020), and such early dementia-related physiological changes could artificially lower NORRISK 2 score and thereby attenuate the observed associations. However, with a follow-up time of 22 years, it is unlikely that this potential bias would be strong enough to impact the results significantly. Third, participation in HUNT4 was lower among individuals with poorer health, raising the possibility of selection bias, which may have resulted in underestimation of the observed associations. Fourth, in a geriatric population with multiple comorbidities, survival bias and the competing risk of death must also be considered. Inclusion of only participants 70 years and older in HUNT4 70+ makes the study prone to survival bias. Further, there may be a missed opportunity to diagnose MCI or dementia before dropout due to death, as people with dementia tend to die earlier than their peers. In addition, participants with a high NORRISK 2 score are more likely to die prematurely from CVD, thus not living long enough to develop cognitive impairment. Fifth, the largely ethnically homogenous composition of the HUNT study may limit the generalizability of the results to people of non-European ancestry. In addition, choosing a risk score intended for a Norwegian population may also limit generalizability to populations that are not closely comparable to the Norwegian population.

The findings of this study contribute to a growing body of evidence indicating that cardiovascular health plays an important role in the development of later life cognitive impairment. By demonstrating that higher CVD risk is associated with both dementia and MCI in a large Norwegian cohort, our results strengthen the evidence that maintaining favorable cardiovascular health throughout adulthood may support healthier cognitive aging. Further, the stronger association observed in females highlights an important sex-specific pattern. Given the high prevalence of dementia among females, these findings underscore the need for further research focusing specifically on dementia in females. Improved understanding of sex-specific pathways may help make more tailored prevention strategies. Overall, the results emphasize the importance of early and sustained efforts to reduce cardiovascular risk as part of a broader dementia-prevention plan and point to the value of incorporating sex-specific perspectives in future research and public health planning.

In conclusion, this study found that the NORRISK 2 score, originally developed to estimate CVD risk, is also associated with dementia and MCI in both males and females, with the strongest association observed in females.

## Data Availability

The data analyzed in this study is subject to the following licenses/restrictions: The Trøndelag Health Study (HUNT) has invited persons aged 13–100 years to four surveys between 1984 and 2019. Comprehensive data from more than 140,000 persons having participated at least once and biological material from 78,000 persons are collected. The data are stored in HUNT databank and biological material in HUNT biobank. HUNT Research Centre has permission from the Norwegian Data Inspectorate to store and handle these data. The key identification in the data base is the personal identification number given to all Norwegians at birth or immigration, whilst de-identified data are sent to researchers upon approval of a research protocol by the Regional Ethical Committee and HUNT Research Centre. To protect participants’ privacy, HUNT Research Centre aims to limit storage of data outside HUNT databank, and cannot deposit data in open repositories. HUNT databank has precise information on all data exported to different projects and are able to reproduce these on request. There are no restrictions regarding data export given approval of applications to HUNT Research Centre. For more information see: http://www.ntnu.edu/hunt/data. Requests to access these datasets should be directed to HUNT Research Center, https://www.ntnu.edu/hunt/.
